# Interleukins 4 and 13 modulate gene expression and promote proliferation of primary human tenocytes

**DOI:** 10.1186/1755-1536-3-9

**Published:** 2010-06-10

**Authors:** Jean-Paul Courneya, Irina G Luzina, Cynthia B Zeller, Jeffrey F Rasmussen, Alexander Bocharov, Lew C Schon, Sergei P Atamas

**Affiliations:** 1 Department of Orthopedics, Union Memorial Hospital, Baltimore, MD, USA; 2 Department of Medicine, University of Maryland School of Medicine and Baltimore VA Medical Center, Baltimore, MD, USA; 3 Department of Chemistry, Towson University, Towson, MD, USA

## Abstract

**Background:**

Tendon disorders (tendinopathies) pose serious biomedical and socioeconomic problems. Despite diverse treatment approaches, the best treatment strategy remains unclear. Surgery remains the last resort because of the associated morbidity and inconsistent outcomes. We hypothesized that, similar to fibroblasts in various organs, tendon fibroblasts (tenocytes) might be responsive to stimulation with interleukins (ILs), particularly IL-4 and IL-13. These two cytokines share sequence homology, receptor chains and functional effects, including stimulation of fibrogenesis. It is unknown whether tenocytes are responsive to stimulation with IL-4 or IL-13. If true, local use of these cytokines might be used to facilitate tendon repair in patients with tendinopathies or used for tendon tissue-engineering approaches to facilitate tenocyte growth on scaffolds in culture.

**Results:**

Tendon tissues that would normally be discarded were obtained during reconstructive surgery procedures performed for clinical indications. Primary tenocytes were derived from Achilles, posterior tibial, flexor digitorum longus and flexor hallucis longus tendon tissue samples. Reverse transcriptase quantitative PCR (RT-qPCR) experiments revealed that mRNAs for the receptor (R) chains IL-4Rα, IL-13Rα1 and IL-13Rα2, but not the common γ-chain were present in all tested tendon tissues and in cultured tenocytes. Levels of IL-13R chain mRNAs were significantly higher than those of IL-4R mRNA. The cultures responded, in a dose-dependent fashion, to stimulation with recombinant human IL-4 or IL-13, by increasing proliferation rates 1.5 to 2.0-fold. The mRNA levels of 84 genes related to cell cycle regulation were measured by RT-qPCR after 6 h and 24 h of activation. The expression levels of several genes, notably CDK6 and CDKN2B changed more than twofold. In contrast to their effects on proliferation, stimulation with IL-4 or IL-13 had little if any effect on the levels of collagen mRNA or protein in cultured primary tenocytes. The mRNA levels of 84 other genes related to extracellular matrix and cell adhesion were also measured by RT-qPCR; expression of only five genes was consistently changed.

**Conclusions:**

Stimulation with IL-4 or IL-13 could be used to facilitate tendon repair *in vivo *or to aid in tendon tissue engineering, through stimulation of tenocyte proliferation.

## Background

Tendon disorders (tendinopathies) are common, and are responsible for much morbidity in sportspersons [1,2], military personnel [3,4] and in the workplace [5,6]. Tendinopathies are associated with trauma, age, male gender, chronic renal or endocrine disease, diabetes [7,8], rheumatoid arthritis [9,10], obesity, steroid therapy, and therapy with fluoroquinolone antibiotics [10,11]. African American or Latino ethnicity increases the risk for major tendon ruptures [3,4]. Tendinopathies of the Achilles tendon (AT) and posterior tibial tendon (PTT) are most common [12]. The mechanisms of tendinopathies are complex, and involve mechanical stress, degenerative changes in the tendon tissue and disorganized healing, along with a contribution from inflammatory processes, although it is unclear [7,13]. The molecular mechanisms of tendinopathies have not been investigated in detail.

The treatment approaches to tendinopathies are diverse, but the optimum treatment remains undetermined, with surgery being the last resort, because of the associated morbidity and inconsistent outcomes [14]. We propose that novel approaches to treating tendinopathies, including postoperative care, should facilitate local tenocyte proliferation and thus strengthen the healing tendon metabolically and mechanically. Tenocytes are collagen-producing mesenchymal cells that make up the majority of cells in a healthy tendon, but unlike fibroblasts in other organs, they express the proteins tenomodulin and scleraxis [15]. Fibroblasts from various organs consistently respond to stimulation with numerous cytokines, by adjusting the rates of proliferation and collagen production [16,17]. Therefore, it is plausible that local administration of cytokines to healing tendons may be beneficial in facilitating tenocyte proliferation and thus the overall tendon repair. Additionally, fibroproliferative cytokines may prove useful in tendon tissue-engineering approaches, to facilitate growth of primary human (including autologous) tenocytes on artificial scaffolds, for subsequent use as implants during reconstructive tendon surgeries.

Interleukin (IL)-4 and IL-13 are prototypic immunomodulatory T-helper (Th)2 cytokines known to have profibrotic effects [16-18]. These two cytokines share sequence homology and cell surface receptor chains, including IL-4Rα, IL-13Rα1, IL-13Rα2 and the common gamma chain (γ_c_). They also share numerous immunomodulatory effects and, relevant to our study, effects on proliferation and gene expression in fibroblasts of diverse tissue origin [16-18]. We hypothesized that primary human tenocytes might be responsive to the profibrotic effects of these two cytokines and thus might be used locally to promote tendon healing in patients with tendinopathies or for tendon tissue-engineering applications. To begin addressing this hypothesis, we investigated whether human tendon tissues and primary tenocytes express IL-4/IL-13 receptor chains. We also assessed the effects of recombinant human (rh)IL-4 and rhIL-13 on cultured human tenocytes.

## Materials and methods

### Patients and tendon tissue samples

Patients with tendinopathies of the AT or PTT were enrolled in this study. All procedures were reviewed and approved by the MedStar Research Institute and the University of Maryland Institutional Review Boards. Informed consent was obtained from all patients.

Tendon tissues that would otherwise be discarded were obtained from normal and injured/diseased tendons during reconstructive surgery procedures performed for clinical indications. These tissues included the tendinopathic portion of the PTT or AT, and the healthy (non-tendinopathic) portion of the flexor digitorum longus (FDL) tendon, the flexor hallucis longus (FHL) tendon or the AT. The diseased sections of the tendon were identified by the characteristic thickening of the outer diameter, fissuring, surface irregularity, fibrillation, and a more gelatinous consistency and yellowish discoloration compared with normal tendons; some of the diseased tendons were also ruptured or attenuated. The specific numbers of patients involved in each experiment are indicated in the Results and the figure legends.

### Primary tenocyte explant cultures

Separate primary cell cultures were established from healthy and diseased portions of tendon tissues obtained from each patient. Harvested tendon tissues were used either for mRNA purification or for primary explant cell cultures. For cell culture, the tissues were rinsed twice in phosphate-buffered saline containing 1% antibiotic mixture (100 μg/ml penicillin and 100 μg/ml streptomycin), cut into 1 mm^3 ^pieces under sterile conditions, and digested with 0.25% trypsin (w/v) in ethylenediaminetetraacetic acid (EDTA) for 5 min. The digested tissues were transferred to 35-mm culture dishes and incubated in 5% CO_2_/95% air at 37°C with Dulbecco's modified Eagle's medium/nutrient mix F-12 (DMEM/F12) medium containing 10% fetal bovine serum and 1% antibiotic mixture as described above. The culture medium was changed twice a week. Emerging cells were split by trypsinization, seeded into 75-cm^2 ^culture flasks at 1 × 10^5 ^cells per flask, and cultured in the same high serum (10%) cell culture medium, until they were split again after reaching approximately 1 × 10^6 ^cells per flask. Tenocyte identity was confirmed by assessing the expression of a tenocyte-specific gene (scleraxis) and genes for collagens α1(I), α2(I) and α1(III) in real-time PCR assays with specific primers. Cells were used for experiments at passages 3 to 5. For experiments, cells were trypsinized, seeded into six-well plates at 2.5 × 10^5 ^cells/well or 96-well plates at 2.5 × 10^3 ^cells/well, and incubated overnight in dialyzed low serum (0.5%) cell culture medium. All experiments were then performed in the same low serum medium. All cell culture reagents were from Invitrogen (Carlsbad, CA, USA).

Cultured tenocytes were stimulated with rhIL-13 or rhIL-4, which were purchased from R&D Systems (Minneapolis, MN, USA). Changes in cell proliferation rates were assessed in quadruplicate, using a cell proliferation assay (CellTiter Aqueous; Promega, Madison, WI, USA) in accordance with the manufacturer's recommendations, as previously described [18]. The assays were calibrated with titrated known amounts of tenocytes.

Changes in gene expression were assessed in real-time quantitative (q)PCR assays as described below. Western blotting assays for type I collagen levels in tenocyte cell cultures were performed and assessed by densitometry of the specific protein bands as previously described [19].

### Reverse transcriptase qPCR

To obtain mRNA, the tendon tissue samples were immediately sectioned into pieces < 3 mm^3 ^in size, frozen in liquid nitrogen, and pulverized under liquid nitrogen with mortar and pestle. The tissue pellet was transferred into a glass tube homogenizer where it was further homogenized in TRIzol reagent (Invitrogen, Carlsbad, CA, USA). Total mRNA was purified from the processed tendon tissues or from cultured tenocytes using TRIzol, and reverse transcription performed (SuperScript II Reverse Transcriptase; Invitrogen) in accordance with the manufacturer's recommendations. Purification of mRNA from cultured primary cells was also performed using TRIzol as recommended. The resulting cDNA samples were used for real-time qPCR using the following three approaches, in all of which the 2^-ΔΔCt ^method was used to assess the amplitude of changes in gene expression induced by IL-13 and IL-4.

The initial pilot experiments were performed on cDNA samples from total human tendon tissues in TaqMan assays with specific primers and probes that were designed based on GenBank sequences for human IL-4Rα or IL-13Rα2 using Primer Express V.2.0 software (Applied BioSystems, Foster City, CA, USA) and synthesized by Integrated DNA Technologies (Coralville, IA, USA). Primers and TaqMan probe for glyceraldehyde-3-phosphate dehydrogenase (GADPH) (Applied BioSystems) were used as positive controls. PCR assays were performed in a thermal cycler (ABI PRISM 7000; Applied BioSystems), with all samples tested in triplicate for each target.

Experiments on cultured tenocytes measured expression levels of scleraxis, IL-13Rα1, IL-13Rα2, IL-4Rα, γ_c_, and the collagen chains COL1A1, COL1A2 and COL3A1. As a reference sequence, 18S rRNA was used. All pre-validated primers and chemistry for SYBR Green detection were from SABiosciences (Frederick, MD, USA). The lack of primer dimers and the specificity of amplification were further confirmed by agarose gel electrophoresis of PCR products, which all gave a single band of expected size, and by automated sequencing of PCR products. Thermal cycling was performed using a real-time PCR system (StepOnePlus; Applied BioSystems), and all samples were tested in triplicate for each target.

Finally, quantitative profiling of genes related to cell cycle regulation or, separately, connective tissue, was performed using the RT^2 ^qPCR System (SABiosciences) and the StepOnePlus system. This approach allowed simultaneous real-time qPCR analyses of the mRNA levels of 84 relevant genes, normalized to five different reference targets (β2-microglobulin, hypoxanthine phosphoribosyltransferase 1, ribosomal protein L13a, GAPDH and β-actin). Combined tenocytes derived from the AT of two separate donors were stimulated with 150 ng/ml rhIL-13 or rhIL-4, each on two separate occasions, for 6 h or 24 h as indicated, and RT-qPCR analyses performed. To be selected, genes had to have a cytokine-stimulated increase or decrease in gene expression that was consistently equal to or greater than twofold in both IL-13- and IL-4-stimulated cultures compared with non-stimulated controls in each independent stimulation. In addition, the absolute level of expression for each gene was required to be sufficient, as judged by amplification occurring earlier than an arbitrarily established threshold of 32 cycles of PCR amplification.

## Results

### Primary tenocytes express IL-13R and IL-4R chains

**Figure 1:**
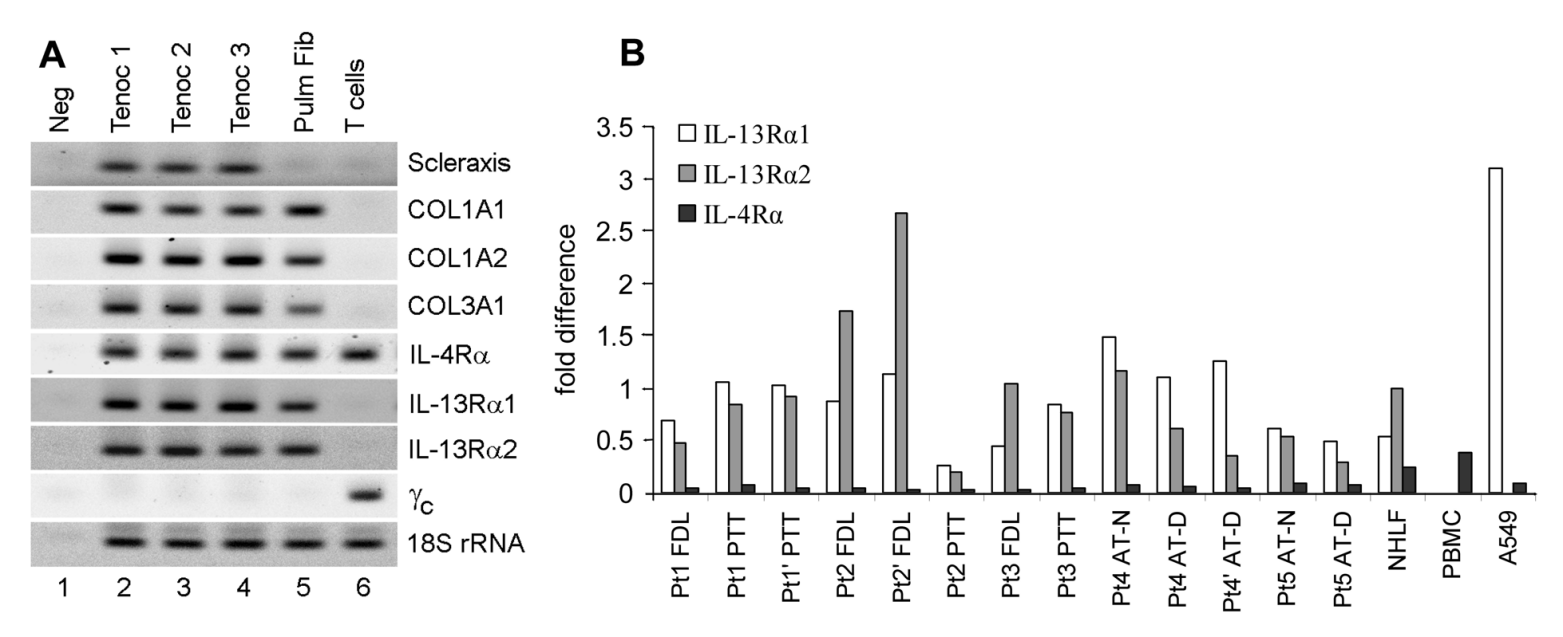
**Reverse transcriptase quantitative (q)PCR analyses of interleukin (IL)-4 receptor (R)/IL-13R mRNA expression **. **(a) **Ethidium bromide gels of PCR products after 35 cycles of qPCR with cDNAs from cultured indicated cells with indicated primers. Tenocytes (Tenoc) expressed scleraxis mRNA, confirming the phenotypic identity of these cells. Tenocytes and pulmonary fibroblasts but not T cells expressed mRNA for collagen chains. All tested cell types expressed IL-4Rα mRNA, and all cell types except T cells expressed IL-13Rα1 and IL-13Rα1 mRNAs. Only T cells expressed common gamma chain (γc). **(b) **Quantitative analyses reveal relative expression of receptor chains in cultured tenocytes from indicated tendons of five separate donors (SYBR Green-based quantification, normalized to 18S rRNA). The prime marks indicate separate cultures developed at a different time from the same tissue source and analyzed at a different passage. A549 = transformed pulmonary epithelial cell line; NHLF = normal human lung fibroblasts, PBMC = peripheral blood mononuclear cells from a healthy volunteer.

Initial pilot RT-qPCR experiments assessed whether mRNAs for IL-4Rα and IL-13Rα2 might be expressed in whole tendon tissues from nine independent donors (Table 1). These assays tested PTT and FDL tissues from six donors, FHL tissue from one donor and AT tissues from three donors (Table 1). In most cases, expression levels of IL-4Rα and IL-13Rα2 mRNAs were similar to those of the ubiquitous GAPDH mRNA; an irrelevant target IL-6 amplified much later in the cycle, suggesting substantially lower levels of IL-6 mRNA in whole tendon tissues (Table 1). We considered that the surgically obtained whole tendon tissues might be contaminated with blood or adjacent non-tendon tissues, therefore subsequent experiments were performed on cultured tenocytes. RT-qPCR experiments revealed that cultured primary tenocytes but not pulmonary fibroblasts or T cells expressed scleraxis mRNA (Figure 1a), thus confirming the phenotypic identity of these cells as tenocytes. Tenocytes and pulmonary fibroblasts but not T cells expressed mRNAs for collagen chains (Figure 1a). Note that all tested mesenchymal cells expressed IL-4Rα, IL-13Rα1 and IL-13Rα2 chains, but only T cells expressed the γ_c _in combination with IL-4Rα chain. Quantitative analyses (Figure 1b) revealed that tenocytes express higher levels of IL-13R chains compared with IL-4Rα. These observations suggested that tenocytes might be responsive to stimulation with IL-13 or IL-4, as these two cytokines are known to share receptor chains. Therefore, tenocytes were stimulated with rhIL-13 or rhIL-4 in the subsequent experiments.

**Table 1:** Real-time PCR values for indicated target genes in mRNA purified from tendon tissue samples.

Patient	Tendon tissue	State	**C**_**t **_**, mean ± SD**^**a**^			
			
			GAPDH	**IL-4R**α	**IL-13R**α**2**	IL-6
1	FDL	Healthy	23.3 ± 0.3	25.8 ± 0.6	26.1 ± 0.1	32.1 ± 0.1
	PTT	Diseased	23.8 ± 0.2	24.7 ± 0.1	24.4 ± 0.4	32.2 ± 0.1
2	FDL	Healthy	27.5 ± 0.7	32.4 ± 0.5	33.2 ± 0.5	37.0 ± 0.6
	PTT	Diseased	24.7 ± 0.5	26.7 ± 0.1	25.6 ± 0.6	37.7 ± 0.9
3	FDL	Healthy	25.0 ± 0.4	30.0 ± 0.8	31.3 ± 0.7	34.1 ± 0.9
	PTT	Diseased	26.1 ± 0.5	29.6 ± 0.6	21.8 ± 0.8	35.0 ± 0.8
4	FHL	Healthy	26.5 ± 0.3	27.1 ± 0.2	ND	38.8 ± 0.8
	AT	Diseased	27.3 ± 0.1	30.0 ± 0.3	ND	U
5	FDL	Healthy	27.9 ± 0.6	30.2 ± 0.5	31.1 ± 0.5	36.6 ± 0.7
	PTT	Diseased	28.4 ± 0.7	30.0 ± 0.6	31.7 ± 0.7	35.8 ± 0.6
6	FDL	Healthy	29.2 ± 0.9	32.4 ± 0.8	31.3 ± 0.7	U
	PTT	Diseased	30.1 ± 0.9	32.2 ± 0.8	32.1 ± 0.9	U
7	FDL	Healthy	29.1 ± 0.7	ND	28.8 ± 0.6	36.8 ± 0.9
	PTT	Diseased	28.6 ± 0.6	ND	26.3 ± 0.5	37.0 ± 0.9
8	AT	Healthy	25.5 ± 0.2	27.4 ± 0.4	27.5 ± 0.3	35.4 ± 0.8
		Diseased	20.2 ± 0.1	22.7 ± 0.3	24.5 ± 0.3	34.4 ± 0.7
9	AT	Healthy	26.5 ± 0.4	29.3 ± 0.5	U	36.2 ± 0.7
		Diseased	26.5 ± 0.3	25.9 ± 0.3	25.9 ± 0.6	31.0 ± 0.6

^a^Each cDNA sample was tested in triplicate.

AT = Achilles tendon; FDL = flexor digitorum longus; FHL = flexor hallucis longus; ND = no data (not tested); PTT = posterior tibial tendon; U = undetermined (no amplification up to PCR cycle 40).

### Primary tenocytes proliferate in response to stimulation with IL-13 or IL-4

**Figure 2:**
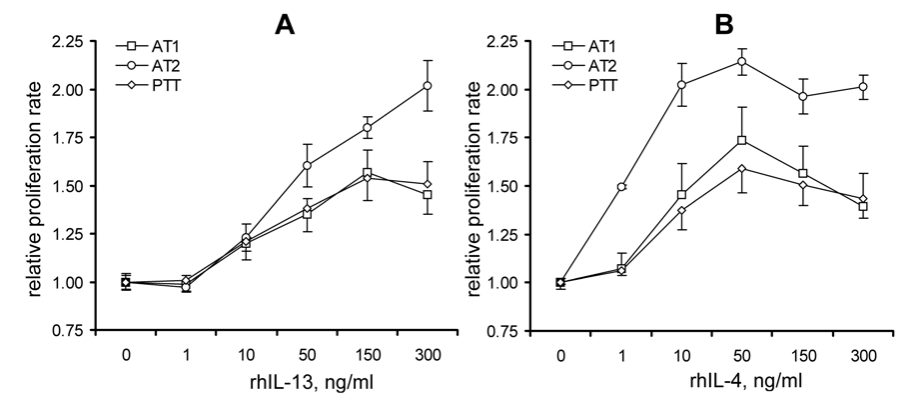
**Effects of IL-13 and IL-4 on proliferation of primary tenocytes **. Stimulation with **(a) **IL-13 or **(b) **IL-4 accelerated proliferation of primary tenocytes in a dose-dependent fashion (CellTiter Aqueous assays, day 7). Data show fold increase in proliferation rate ± SD, in tenocytes derived from indicated tendon types.

Stimulation with rhIL-13 or rhIL-4 leads to dose-dependent increases in tenocyte proliferation rates (Figure 2). One-way ANOVA analyses revealed that the increases were significant at cytokine concentrations of 10 to 300 ng/ml. Note that in both cases, the increase in proliferation rate was between 1.5- and 2-fold at a concentration of 150 ng/ml. The effect of IL-13 remained approximately the same or was further increased at 300 ng/ml, whereas the maximum effect of IL-4 was observed at 50 ng/ml, with a gradual decline at 150 ng/ml and 300 ng/ml. In addition to the two AT tenocytes cultures and one PTT culture shown in Figure 2, we tested the effect of a single concentration of 150 ng/ml IL-13 or IL-4 on four other PTT and four FDL tenocyte cultures. There were no differences in-fold increases of proliferation rates induced by IL-13 or IL-4, or between PTT or FDL tenocyte cultures (*P *> 0.05, two-tailed Student's *t*-test). The average increase in proliferation rates at this single concentration of either IL-13 or IL-4 was 1.75 ± 0.27-fold compared with non-stimulated control cultures.

To identify the molecular pathway through which IL-13 and IL-4 may regulate tenocyte proliferation, we used RT-qPCR to assess changes in the expression level of 84 molecules known to be involved in the cell-cycle regulation. We combined equal amounts of tenocytes derived from the ATs of two volunteers, stimulated them with rhIL-13 or rhIL-4 for 6 and 24 hrs on two independent occasions, and performed RT-qPCR and analyzed gene expression as described in Methods. The expression levels of only four genes were changed at 6 h, but those of 16 genes were changed at 24 h, with only two genes (CDK6 and CDKN2B) consistently changed at 6 h and 24 h (Table 2). It is likely that these pathways mediate the effects of IL-13 and IL-4 on tenocyte proliferation.

**Table 2:** Average fold differences in steady-state mRNA levels compared with non-stimulated tenocytes, at 6 h and 24 h of activation, for cell cycle-related genes.

mRNA	6 h		24 h	
	
	IL-4	IL-13	IL-4	IL-13
CDK6	2.22	2.26	3.08	2.62
CDKN2B	0.31	0.32	0.22	0.28
CDKN1A	2.21	2.25	--	--
Nibrin	2.97	3.04	--	--
Cyclin B1	--	--	0.35	0.33
Cyclin B2	--	--	0.23	0.21
Cyclin E1	--	--	3.95	3.97
CDK1	--	--	0.17	0.14
CDK5	--	--	6.35	2.1
CDKN3	--	--	0.16	0.19
Cdc20	--	--	0.24	0.27
Cdc21	--	--	2.6	2
Cdc46	--	--	2.78	1.91
GADD45A	--	--	0.37	0.46
HUS1^a^	--	--	2.72	1.9
Ki-67^b^	--	--	0.15	0.2
Dp-1^c^	--	--	2.05	3.64
p53^d^	--	--	2.63	3.17

For clarity, the standard deviations are not shown, but the variability within each group did not exceed 0.25 cycles of PCR amplification.

-- = difference was > 0.5 but < 2.0; Cdc = cell division cycle; CDK = cyclin-dependent kinase; CDKN = cyclin-dependent kinase inhibitor; GADD45A = growth arrest and DNA-damage-inducible alpha.

^a^Checkpoint homolog.

^b^Proliferation-related antigen.

^c^Transcription factor.

^d^Tumor suppressor protein.

### The effect of IL-13 and IL-4 on collagen production by cultures tenocytes is inconsistent

We then assessed whether IL-13 or IL-4 can induce production of collagen in cultured primary AT, PTT or FDL tenocytes. AT tenocytes were derived from separate healthy or diseased tendon sections taken from the same donor. PTT and FDL tenocytes were from a separate patient, and were tested on two independent occasions. Steady-state levels of COL1A1, COL1A2 and COL3A1 mRNAs were assessed at 0, 1.5, 3, 6 and 24 hrs of stimulation with 50, 150 or 300 ng/ml rhIL-13. The-fold difference between stimulated and non-stimulated tenocytes cultures varied from 0.8 to 1.3, and did not reach significance in any instance (*P *> 0.05, one-way ANOVA).

Western blotting assays assessed production of type I collagen protein in separate primary AT tenocyte cultures derived from healthy and diseased sections of the tendons, each from three different patients, and in a primary FHL tenocyte culture from a fourth patient. Each experiment was repeated on two independent occasions. The changes in the density of collagen bands varied from 0.9 to 1.2-fold in IL-13-stimulated samples compared with non-stimulated controls (*P *> 0.05, one-way ANOVA). By contrast, stimulation of tenocytes with 1 ng/ml of recombinant human transforming growth factor-β (used as a positive control) induced a 3.3 ± 0.2-fold increase in the density of the collagen band (*P *< 0.05, two-tailed Student's t-test, data not shown).

Based on these observations, we concluded that IL-13 has minimal, if any, effect on collagen production in cultured tenocytes. To further assess whether IL-13 or IL-4 might have any effect on other extracellular matrix (ECM)-related genes, RT-qPCR (RT^2 ^real-time PCR System; SABiosciences) was used to measure gene expression for 84 genes related to the ECM, with results normalized to those from five housekeeping genes. In these experiments, AT tenocytes from two volunteers were pooled and stimulated with 150 ng/ml of IL-4 or IL-13 for 6 h, each on two independent occasions, and the gene expression was tested by RT-qPCR. Again, there were no significant changes in the expression of collagen type I or type III chain genes, nor were there consistent changes in the expression of the majority of tested genes within two-fold differences. Only five genes (Table 3) met the criteria described in the Methods section (consistent significant change induced by both IL-13 and IL-4).

**Table 3:** Average fold differences in steady-state mRMA levels compared with non-stimulated tenocytes, at 6 h of activation, for extracellular matrix- and cell adhesion-related genes.

mRNA	IL-4	IL-13
VCAM1	15.88	27.38
Thrombospondin 1	3.92	2.81
Tenascin C	2.66	2.94
ADAMTS	0.21	0.29
Integrin β5	0.22	0.30

For clarity, the standard deviations are not shown, but the variability within each group did not exceed 0.25 cycles of PCR amplification.

-- = difference was > 0.5 but < 2.0; ADAMTS = A disintegrin-like and metalloproteinase with thrombospondin type 1 motif; VCAM = vascular cell adhesion molecule.

## Discussion

We found that primary human tenocytes derived from healthy tendon tissues and tendon segments involved in tendinopathy express the IL-13R and IL-4R chains except for γ_c _(Figure 1). They respond to stimulation with rhIL-13 or rhIL-4 by increasing proliferation (Figure 2) but not collagen production, and by changing expression of several genes, including those for cell cycle-related factors (Table 2) and ECM- and cell adhesion-related factors (Table 3). Thus, IL-4 and IL-13 might have therapeutic potential as tendon-healing agents used locally; the systemic use of these immunomodulatory cytokines is not advisable because of the danger of side effects. These cytokines might also be useful in future tendon tissue-engineering applications to facilitate tenocyte growth on artificial scaffolds for subsequent use in reconstructive tendon surgery.

Stimulation with IL-4 or IL-13 induced changes in expression of several cell cycle-related genes, particularly CDK6 and CDKN2B, which were consistently changed at 6 h and 24 h of stimulation (Table 2). Further mechanistic studies will address the role of each of these factors, with the ultimate goal of developing novel approaches to fine-tune tenocyte proliferation. Neither IL-4 or IL-13 had any effect on collagen mRNA or protein levels or on the expression of the majority of 84 tested connective tissue- and cell adhesion-related genes, with a few exceptions (Table 3). Therefore, these two cytokines might facilitate tendon repair through their tenoproliferative effects.

Other researchers have already addressed an adjacent but different hypothesis, by studying the role of germline deficiency of IL-4 in otherwise healthy mice [20] and in a mouse model of tendon healing [21]. The results [20,21] were inconclusive, probably due to numerous acknowledged limitations of their study design, and there were substantial differences between the study designs of those previous reports and our current study. Those studies used a mouse model, whereas we investigated primary human tendon tissues and tenocytes, thus our results are more likely to be relevant to human health. Their experiments addressed the importance of natural systemic levels of IL-4 in mice on tendon development, homeostasis and healing; such natural levels are notoriously low in the absence of allergy or other Th2 activation. By contrast, our investigation focused on the therapeutic potential of high doses of locally administered IL-4 or IL-13. Moreover, the previous studies did not address a potential role of IL-13, which shares numerous functions with IL-4, including effects on proliferation and gene expression in tenocytes (Figure 2, Table 1, Table 2). Because the mice in the previous reports [20,21] were deficient in IL-4- but not IL-13, it is logical to expect that IL-13 would significantly compensate for the lack of IL-4 in those animal models. Therefore, our study provides novel findings of potential applied significance.

In our study, the expression of the IL-4R and IL-13R chains and the effects of IL-4 and IL-13 were consistent in tenocytes derived from various donors, various tendon types, and from both healthy and diseased tendon tissues. All the PTT tissues in this study were from diseased segments of the tendon and all FDL tissues were from healthy segments, as autologous FDL was used for surgical PTT reconstruction in the same patients. AT tissues were also tested from healthy and diseased segments of the tendon. Therefore, local use of rhIL-4 and/or rhIL-13 might be broadly applicable for therapeutic facilitation of tendon healing.

One possible weakness of our study is that the increase in proliferation rates of tenocytes did not exceed two-fold, which is relatively modest compared with the effects of IL-4 on the proliferation rates of immune cells, such as lymphocytes. However, it is important to keep in mind that lymphocytes proliferate rapidly upon stimulation but also undergo rapid apoptosis upon completion of the immune response. By contrast, tenocytes are ECM-producing connective tissue cells, and large-amplitude changes in connective tissue would have far-reaching structural and functional consequences. It is, therefore, plausible that the amplitude of changes in this study was consistent with previous observations in fibroblasts from tissues other than tendons that respond to stimulation with IL-4 or IL-13 with similar amplitude [16-18]. Thus, the results of this study are relevant and significant.

The biological purpose for the receptor expression and responsiveness to IL-4 and IL-13 in primary tenocytes is not clear. These two cytokines are commonly involved in allergic and antiparasitic immune responses. However, it would be difficult to imagine such immune reactions in the tendon, which is very compact, highly fibrotic tissue, whose sheer density prevents the degree of swelling and immune cell extravasation commonly seen in other tissues. Therefore, it is unlikely that tendon tissue would contain numerous IL-4 or IL-13-producing cells (such as T cells) or participate in classic immune reactions [13]. We did not observe any IL-4 or IL-13 mRNA production in tendon tissues or cultured primary tenocytes by RT-qPCR (data not shown). We also performed experiments in which primary AT and PTT tenocytes at passage 3 were stimulated with 100 ng/ml of either rhTNF-α, rhIL-1β or rhIFN-γ for 6 h or 24 h, with levels of IL-4 and IL-13 mRNAs measured by RT-qPCR in these samples. No PCR product was detected for IL-4 or IL-13 mRNA before or after stimulation with rhTNF-α, rhIL-1β or rhIFN-γ at these times. In the same experiments, the IL-4 and IL-13 mRNA targets were readily detectable in the same amount of total RNA (as judged by similar RT-qPCR amplification of an 18s rRNA reference target) from purified primary T cells (positive control, data not shown). However, other authors have recently reported that another interleukin, IL-10, is also biologically active in tenocytes [22,23], further suggesting that primary tenocytes do proliferate and change gene expression in response to immunomodulatory cytokines, thus this feature might be useful for biomedical applications.

## Conclusions

Primary human tenocytes derived from healthy and diseased tendon tissue express IL4Rα, IL-13Rα1 and IL-13Rα2 chains but not the γ_c _chain. Both rhIL- 4 and rhIL-13 stimulate tenocyte proliferation and regulate expression of several genes, including genes whose protein products control the cell cycle; however, neither cytokine consistently regulates production of collagen in primary tenocytes. These observations suggest that local administration of IL-4 and/or IL-13 to the healing tendon might be beneficial in accelerating recovery in patients with tendinopathies, without affecting systemic regulation of inflammation or immunity by these cytokines. These cytokines might be also used in tendon tissue-engineering applications, to facilitate the growth or primary, including autologous, tenocytes on scaffolds, to be used as implants in reconstructive tendon surgery.

## Competing interests

The authors had no competing interests that might affect experimental data, data analyses or interpretations or conclusions in this study.

## Authors' contributions

All authors have read and approved the manuscript and contributed to the study design, data acquisition and analysis, interpretation of the data, and drafting and revision of the manuscript. JPC, LCS and SPA had full access to all of the data in the study and take responsibility for the integrity of the data and the accuracy of data analysis. The study was designed by LCS and SPA. Data were acquired and partially analyzed by JPC, IGL, CBZ, JFR and LCS. Complete data analyses and interpretations were performed by JPC and SPA. Statistical analyses were performed by SPA. The manuscript was prepared by JPC and SPA.
